# Supplementary figures and images for: Integrative genomic and transcriptomic analyses of a bud sport mutant ‘Jinzao Wuhe’ with the phenotype of large berries in grapevines
Source: PeerJ. 2023 Jan 3;11:e14617. doi: 10.7717/peerj.14617 (PMC9817954; doi:10.7717/peerj.14617)

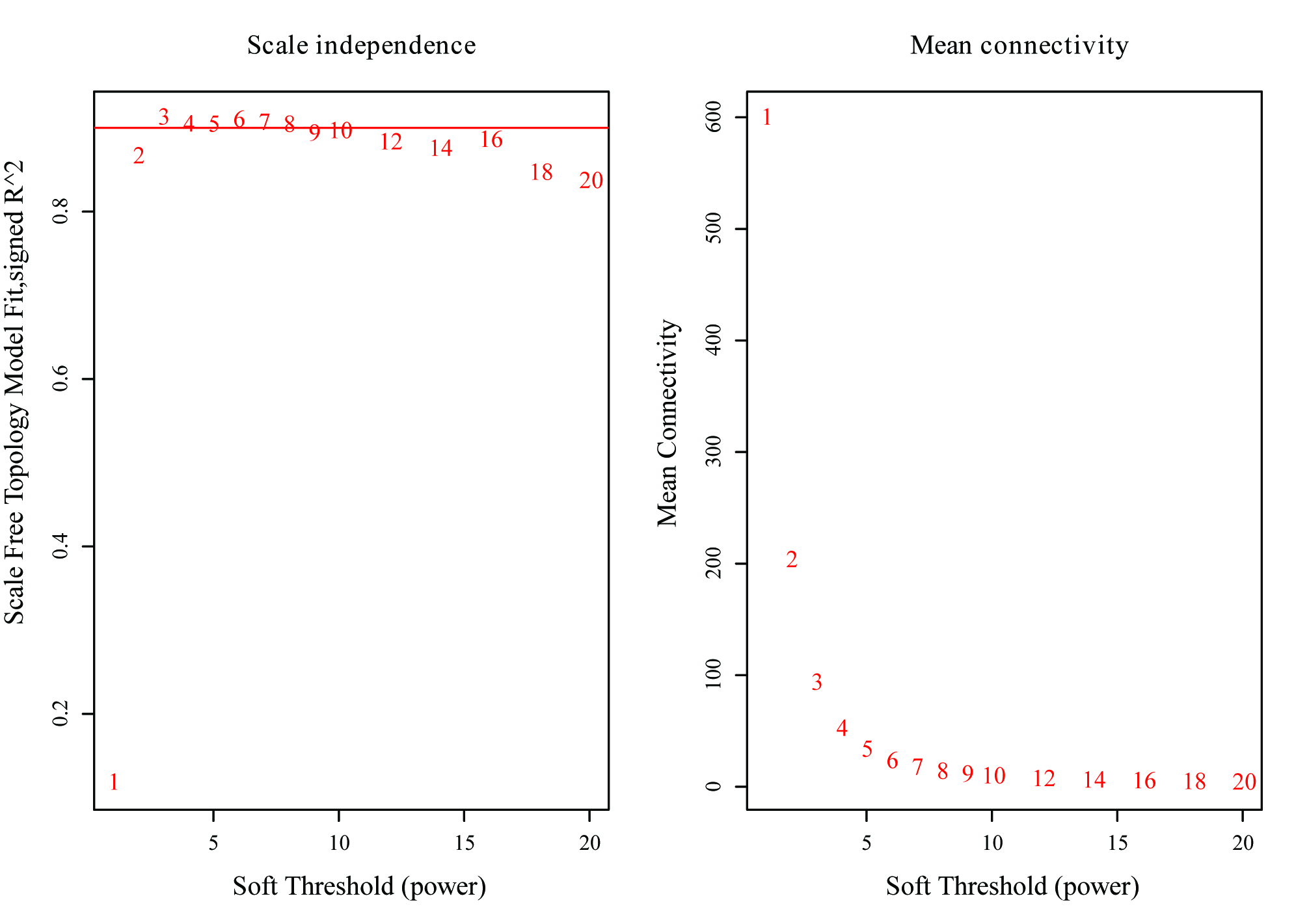

Supplement: Supplemental Information 1 [file peerj-11-14617-s001.tif]
